# Metagenomic Analysis Reveals the Heterogeneity of Conjunctival Microbiota Dysbiosis in Dry Eye Disease

**DOI:** 10.3389/fcell.2021.731867

**Published:** 2021-11-25

**Authors:** Qiaoxing Liang, Jing Li, Yanli Zou, Xiao Hu, Xiuli Deng, Bin Zou, Yu Liu, Lai Wei, Lingyi Liang, Xiaofeng Wen

**Affiliations:** ^1^ State Key Laboratory of Ophthalmology, Guangdong Provincial Key Laboratory of Ophthalmology and Visual Science, Zhongshan Ophthalmic Center, Sun Yat-sen University, Guangzhou, China; ^2^ Department of Ophthalmology, Foshan Hospital Affiliated to Southern Medical University, Foshan, China

**Keywords:** dry eye disease, conjunctival microbiota, metagenomic shotgun sequencing, aqueous tear deficiency, meibomian gland dysfunction

## Abstract

**Background:** Dry eye disease (DED) is a multifactorial inflammatory disease of the ocular surface. It is hypothesized that dysbiosis of the conjunctival microbiota contributes to the development of DED. However, species-level compositions of the conjunctival microbiota in DED and the potential dysbiosis involving microorganisms other than bacteria remain largely uncharacterized.

**Methods:** We collected conjunctival impression samples from a cohort of 95 individuals, including 47 patients with DED and 48 healthy subjects. We examined the conjunctival microbiota of these samples using shotgun metagenomic sequencing and analyzed microbial dysbiosis in DED at the species level.

**Results:** The conjunctival microbiota in DED exhibited a decreased α-diversity and an increased inter-individual variation. The α-diversity of female patients with DED was higher than that of male patients. Despite a decreased prevalence in DED, 23 microbial species were identified to show abnormally high abundance in DED samples positive for the species. Among these species, a fungal species *Malassezia globosa* was enriched female patients. In addition, distinct patterns of associations with disease status were observed for different species of the same genus. For DED subtypes, *Staphylococcus aureus* and *S. capitis* were associated with meibomian gland dysfunction (MGD), whereas *S. hominis* was enriched in patients solely with aqueous tear deficiency (ATD). The microbiota of patients with a mixed type of diagnosis was more similar to MGD patients than ATD patients.

**Conclusion:** We demonstrated that the conjunctival microbiota dysbiosis in DED is characterized by significant heterogeneity. Microbial signatures may offer novel insights into the complicated etiology of DED and potentially promote the development of personalized treatment for DED in the future.

## Introduction

Dry eye disease (DED) is a multifactorial ocular surface disease with a prevalence ranging from 5 to 50% worldwide (Craig et al., 2017; Stapleton et al., 2017). There are two major types of dry eye, including aqueous deficient and evaporative dry eye (Craig et al., 2017). Aqueous deficient dry eye is featured by decreased tear secretion, whereas evaporative dry eye is featured by increased tear evaporation caused by meibomian gland dysfunction (MGD). The two types of dry eye are not exclusive. A significant number of patients are diagnosed with both aqueous tear deficiency (ATD) and MGD (Tsubota et al., 2020). Therefore, a hybrid form of dry eye (i.e., a mixed type diagnosis of ATD and MGD) has been proposed (Craig et al., 2017).

Emerging evidence suggests that alteration in the ocular surface microbiota is involved in DED (Gomes et al., 2020). However, the majority of prior studies have focused on specific types of DED, such as MGD and Sjögren’s syndrome (de Paiva et al., 2016; Watters et al., 2017; Jiang et al., 2018; Dong et al., 2019; Zhao et al., 2020). In contrast, patients with other types of DED were less represented, especially those with a mixed type of diagnosis. Moreover, most studies have examined the ocular surface microbiota in DED by 16S rRNA sequencing (Jiang et al., 2018; Dong et al., 2019). As a result, microbial dysbiosis at the species level and involving microorganisms other than bacteria remain largely uncharacterized. Most importantly, the DED-associated microbial dysbiosis that was reported by different studies exhibits a high level of inconsistency (Watters et al., 2017; Andersson et al., 2021).

Given the complicated etiology of DED, we hypothesized that the ocular microbial dysbiosis in DED is characterized by significant heterogeneity. The heterogeneity potentially explains the inconsistency between conclusions from previous studies. To test this hypothesis and have a better understanding of the ocular microbial dysbiosis in DED, we surveyed the conjunctival microbiota of patients with DED and healthy individuals using shotgun metagenomic sequencing. We observed the polarization of the abundance of microbial species in DED. In addition, we detected DED-specific sex-related differences in the conjunctival microbiota. Finally, we identified the microbial species signatures of different types of dry eye, including ATD, MGD, and the mixed type of dry eye.

## Materials and Methods

### Participant Recruitment

This study adhered to the tenets of the Declaration of Helsinki. All procedures were performed in compliance with the protocol (#2015MEKY011) approved by the Ethics Committee of Zhongshan Ophthalmic Center, Sun Yat-sen University (Guangzhou, China). Written informed consent was obtained from all participants. Participants were recruited at Zhongshan Ophthalmic Centre from January 2018 through December 2019. DED was diagnosed according to diagnostic criteria proposed by the International Dry Eye Workshop II (Craig et al., 2017). Objective tear film and ocular surface parameters of both eyes were examined, including an evaluation of tear break up time, Schirmer test, meibomian gland and an assessment of subjective symptoms. For DED subtype analysis, patients were classified into the three groups: 1) pure MGD (reduced expressibility and/or quality of the meibum, as well as morphological changes of the lid margins, such as telangiectasia, irregularity and a shifting of the openings of the meibomian glands); 2) pure ATD (Schirmer values ≤ 5 mm/5 min in at least one eye); 3) mixed type, MGD + ATD (both MGD and ATD criteria met).

The exclusion criteria included a history of: 1) topical and systemic anti-bacterial, anti-fungal, or anti-viral treatment within the past 90 days; 2) receiving immunosuppressants within the past 90 days; 3) receiving topical or systemic corticosteroids within one week; 4) any ocular surgery or trauma within the past 6 months; 5) any ocular surface diseases other than dry eye, such as infection, blepharitis, allergic conjunctivitis, Stevens-Johnson syndrome, pterygium, etc.; 6) concurrent systemic diseases; 7) contact lens wearing; 8) smoking; 9) receiving eye drops within the past 90 days.

### Sample Collection

Both eyes of the patients diagnosed with DED were screened and the conjunctival impression sample was collected from the eye with severer symptoms than the other. Before sample collection, the eyes were topically anesthetized with 1−2 drops of alcaine Eye Drop (Alcon, Fort Worth, TX, United States). As previously described (de Paiva et al., 2016; Wen et al., 2017), a sterile semicircle MF membrane filter (REF: HAWP01300, 0.45 l m in diameter; Merck Millipore, Burlington, MA, United States) was placed on the inferior bulbar conjunctiva for 10 s. The membrane was then immediately placed in a sterile tube with 300 µL of Tissue and Cell Lysis Solution (Epicentre, Ambleside, United Kingdom) and stored at −80°C.

### Metagenomic Shotgun Sequencing

DNA was extracted from conjunctival samples using the MasterPure Complete DNA and RNA Purification Kit (Epicentre) according to the manufacturer’s instructions. A total of 100 ng DNA per sample was sonicated into 300–400 bp fragments using Bioruptor (Diagenode, Seraing, Belgium). Sequencing libraries were prepared using the VAHTS universal DNA library Prep Kit for Illumina (Vazyme, Nanjing, China) and quantified by qPCR using the KAPA SYBR FAST qPCR Kit (Kapa Biosystems, Wilmington, MA, United States). Paired-end 2 × 150-bp sequencing was performed on a NovaSeq 6,000 instrument (Illumina, San Diego, CA, United States). Negative blank controls, which had reagents from DNA extraction through sequencing, were processed along with the samples.

### Taxonomic Profiling

Raw sequencing reads were first quality filtered using Trimmomatic (Bolger et al., 2014) v0.36 and PRINSEQ (Schmieder and Edwards, 2011) v0.20.4. Human reads were removed using KneadData v0.6.1 (https://bitbucket.org/biobakery/kneaddata). Filtered reads were mapped using Kraken2 (Wood et al., 2019) v2.0.9 against a custom database composed of 29,943 complete microbial genomes downloaded on May 3, 2020. Complete bacterial, archaeal, and viral genomes were downloaded from the RefSeq database, whereas complete fungal genomes were downloaded from the GenBank database. Taxonomic classification results were filtered using a confidence score of 0.2. Species with more than 10 reads in at least one sample were included in analysis. Contaminant species were detected and removed using the decontam R package (Davis et al., 2018). The most stringent hyperparameter value (p* = 0.5) was applied for frequency-based and prevalence-based contaminant identification of the *isContaminant* function. For the frequency-based method, DNA concentrations were measured by qPCR and obtained during library preparation. The scores from the frequency-based and prevalence-based methods were combined using the “minimum” approach. We further excluded the species whose relative abundance was greater than 0.05% in at least one negative blank control and the species whose relative abundance showed a significant inverse correlation with DNA concentrations (*ρ* < −0.2, *p* < 0.05, Spearman’s correlation) (Jervis-Bardy et al., 2015).

### Statistical Analysis

Statistical analysis was performed using R v4.0.2 software packages. The Shannon diversity index and Bray-Curtis dissimilarity index were computed using the vegan R package. Group differences of the Shannon diversity and Bray-Curtis dissimilarity were analyzed by two-sided Wilcoxon’s rank sum test. Principal coordinates analysis (PCoA) was performed using the ade4 R package. For all boxplots, the box edges denoted the first and third quartiles and the horizontal line denoted the median, with the whiskers extending up to the 1.5-fold interquartile ranges. Associations between disease status and the abundance of microbial species were determined using multivariable regression models implemented in MaAsLin2 (https://huttenhower.sph.harvard.edu/maaslin). Microbial species present in at least 10% samples were included in the association analysis. Transformation and normalization were applied on the microbial species features using default options of MaAsLin2. Transformed abundances were fit with per-feature general linear model (GLM) in which the abundance of each species was modelled as a function of diagnosis (dry eye versus healthy, ATD versus non-ATD, and MGD versus non-MGD) as a categorical variable, age as a continuous covariate, and sex as a binary covariate. For assessment of sex-related differences, the abundance of each species was modelled as a function of sex with age as a covariate.

## Results

### Dysbiosis of Conjunctival Microbiota in DED

**FIGURE 1:**
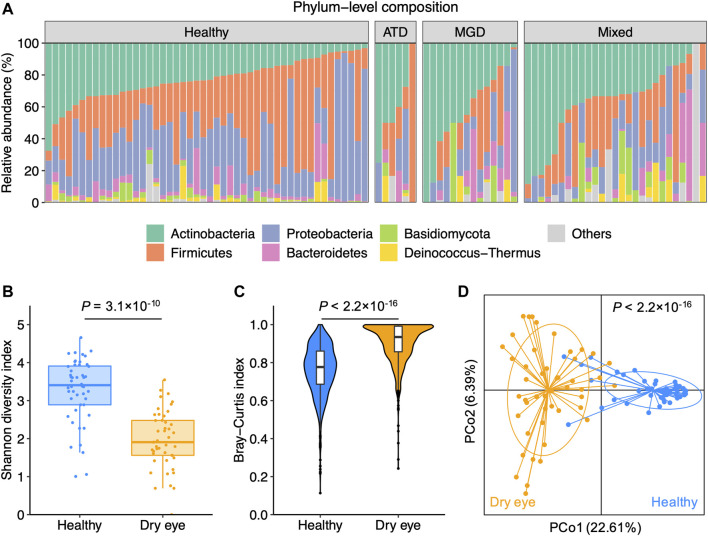
Dry eye is associated with microbial dysbiosis of the conjunctival microbiota **(A)** Phylum-level composition of the conjunctival microbiota of healthy individuals (*n* = 48) and patients with dry eye (*n* = 47). Patients were diagnosed with aqueous tear deficiency (ATD, *n* = 6), meibomian gland dysfunction (MGD, *n* = 14), or a hybrid form of ATD and MGD (mixed, *n* = 27) **(B)** The α-diversity measured with the Shannon index was computed for healthy and dry eye samples **(C)** The β-diversity measured with Bray-Curtis dissimilarity within healthy and dry eye samples **(D)** Principal coordinates analysis of samples from all 95 participants based on the species-level Bray-Curtis distance. *p* values were computed for PCo1 using Wilcoxon’s rank sum test.

The study cohort was composed of 47 patients with DED and 48 healthy individuals (Supplementary Table S1). The group of DED comprised six patients with ATD, 14 with MGD, and 27 with mixed dry eye (Supplementary Table S2). We performed shotgun metagenomic sequencing on the conjunctival impression samples to characterize the taxonomic composition of the conjunctival microbiota. Overall, the phylum-level composition was similar between patients with DED and healthy individuals (Figure 1A). For healthy individuals and patients with different subtypes of DED, Actinobacteria, Firmicutes, Proteobacteria, and Bacteroidetes accounted for the majority of the conjunctival microbiota. Consistent with prior studies (Zilliox et al., 2020; Andersson et al., 2021), the α-diversity of patients with DED was significantly lower than that of healthy individuals (Figure 1B). The β-diversity within patients with DED was higher than that within healthy individuals, suggesting an increased inter-individual variation in the conjunctival microbiota of patients with DED (Figure 1C). We performed the principal coordinates analysis based on species-level composition and observed a clear delineation between DED and healthy samples (Figure 1D). These results demonstrate that DED is associated with microbial dysbiosis of the conjunctival microbiota.

To characterize the microbial species-level alterations related to the microbial dysbiosis, we compared the species profiles between samples from patients with DED and those from healthy individuals. We identified a total of 206 species that were more abundant in healthy individuals (*p* < 0.05). However, we did not detect any species that were significantly enriched in DED (Supplementary Figure S1A). In addition, we found that the majority of microbial species exhibited a decreased prevalence in the DED group in contrast to the healthy group (Supplementary Figure S1B). Specifically, only 50 species were present in at least 10% samples in both the dry eye and healthy groups (Supplementary Figure S1C) and the number decreased to 16 for species present in at least 20% samples (Supplementary Figure S1D). These observations suggest that the dysbiosis of the conjunctival microbiota in dry eye is primarily characterized by the depletion of commensal species.

### Polarization of Microbial Species Abundance in DED

We hypothesized that the lack of general enrichment of microbial species in dry eye might be due to the high level of inter-individual variation in patients with dry eye. To search for the microbial species that contributed prominently to the individuality, we performed principal coordinates analysis on the species-level composition of samples from patients with dry eye. For each species present in at least 10% samples in the dry eye group, we analyzed differences of sample positions in principal coordinate 1 (PCo1) or 2 (PCo2) between the DED samples with the species (denoted as P-DED) and those without the species (denoted as N-DED). We identified 25 species that significantly contributed to either PCo1 or PCo2 (*p* < 0.05). The species list is available in Supplementary Table S3. We found that species of the genera *Streptococcus* and *Corynebacterium* were overrepresented in the list. Other genera that were represented by at least two species included *Cutibacterium*, *Rothia*, and *Staphylococcus*.

**FIGURE 2:**
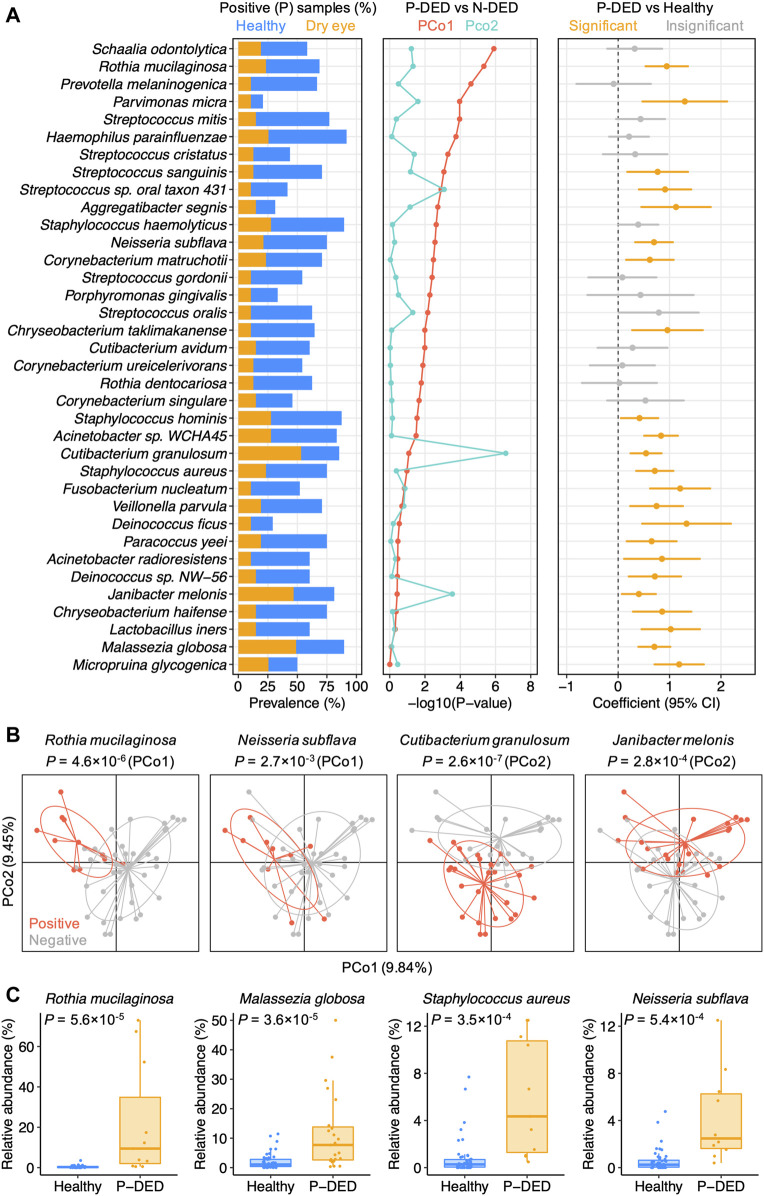
Microbial dysbiosis in dry eye exhibits a high level of heterogeneity **(A)** Microbial species present in at least 10% samples of the dry eye group were shown if they significantly contributed to one of the top two principal coordinates of the dry eye samples or exhibited a polarized distribution in abundance. P-DED, DED samples in which a specific species was detected; N-DED, DED samples in which a specific species was not detected **(B)** Examples of species significantly contributing to PCo1 or PCo2. Samples are colored according to the detection of the species. *p* values were computed using Wilcoxon’s rank sum test **(C)** Examples of species with a polarized distribution in abundance in DED samples compared to healthy samples.

Furthermore, given the dominant decrease in the prevalence of species in patients with DED in contrast to healthy individuals, we compared species relative abundance between P-DED and healthy samples. Intriguingly, we detected 23 species that were significantly enriched P-DED samples (*p* < 0.05). The species list is available in Supplementary Table S4. Notably, 46 out of 47 samples from patients with DED contained at least one of the 23 species. Among these species, 12 species significantly contributed to either PCo1 or PCo2 (Figure 2A). Examples of such species were shown in Figure 2B.

Of note, despite the absence in a part of patients with DED, the first quartiles of relative abundance of the microbial species enriched in P-DED samples were generally higher than the third quartiles of that in samples from healthy individuals (Figure 2C). This finding implies a polarized distribution of the abundance of microbial species in the conjunctival microbiota of patients with DED compared with healthy individuals. In other words, some microbial species might exhibit a relatively even distribution in healthy individuals, whereas they might be either depleted or with abnormally high abundance in patients with DED. Collectively, these results highlight the heterogeneous microbial dysbiosis in dry eye.

### Distinct Sex-Related Differences in Conjunctival Microbiota

**FIGURE 3:**
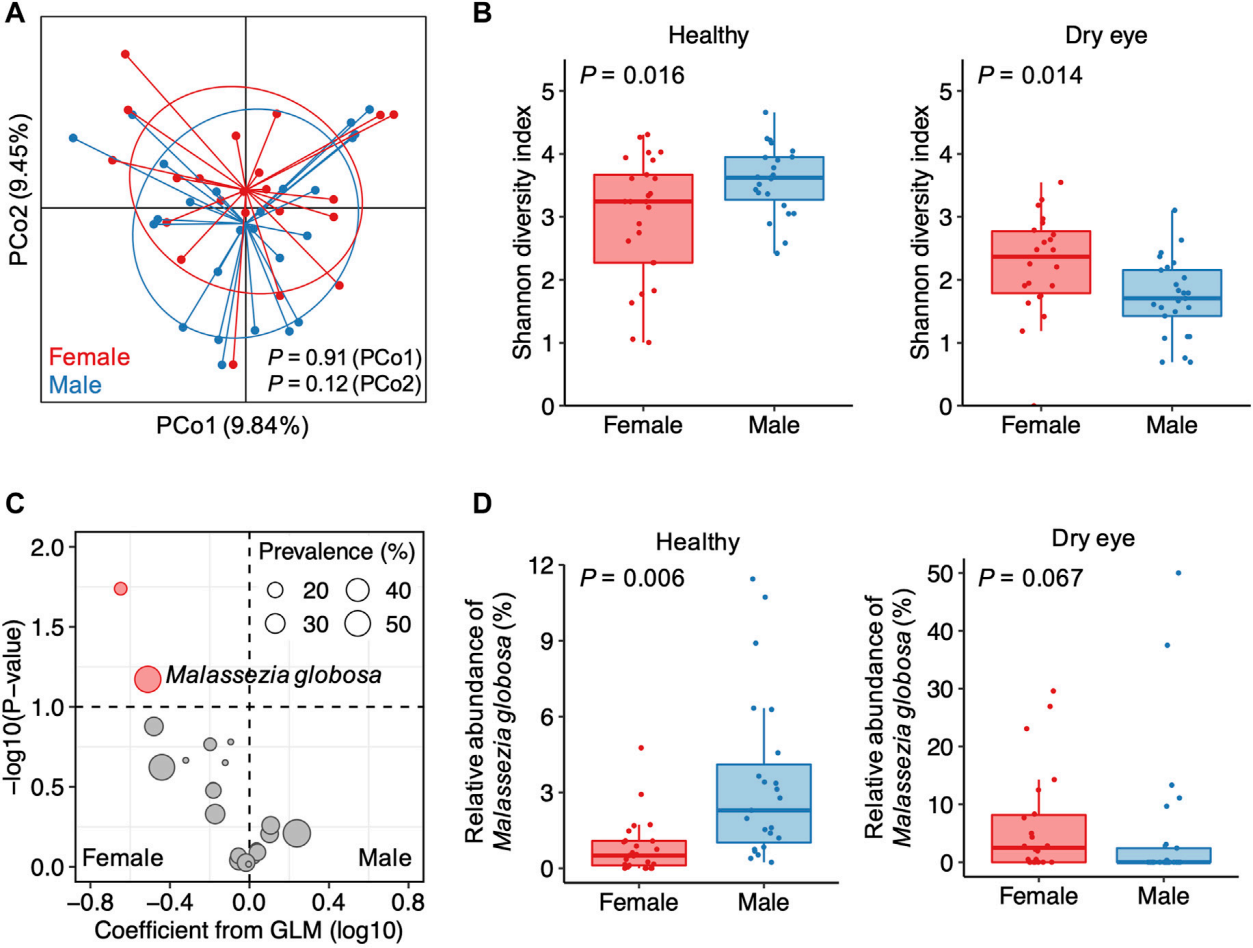
Sex-related differences in the conjunctival microbiota of patients with dry eye **(A)** Principal coordinates analysis of samples from patients with dry eye. Samples are colored according to the sex of patients. *p* values were computed using Wilcoxon’s rank sum test **(B)** Differences in the α-diversity between female and male individuals in the healthy and dry eye groups, respectively **(C)** The volcano plot demonstrating associations of the 23 species with polarized abundance with sex. Sizes of dots reflect their prevalence in the dry eye group. GLM, general linear model **(D)** Differences in the relative abundance of *Malassezia globosa* between female and male individuals in the healthy and dry eye groups, respectively.

With a higher DED prevalence in women than men, female sex is considered as a risk factor for the development of DED (Sullivan et al., 2017). We therefore assessed whether sex factors are associated with the heterogeneity of the conjunctival microbiota in DED. Overall, sex was a minor factor accounting for inter-individual variation in DED (Figure 3A). We compared the α-diversity and the relative abundance of microbial species between female and male individuals in the healthy and dry eye groups, respectively, using multivariable regression models with age as a covariate. Consistent with previous observations (Ozkan et al., 2017), males harbor a more diverse conjunctival microbiota than females in healthy individuals (*p* = 0.016; Figure 3B). Unexpectedly, the α-diversity of female patients with DED was higher than male patients (*p* = 0.014).

Due to a major depletion of microbial species in DED, a few species were solely enriched in males for healthy individuals (Supplementary Figure S1A, B). For patients with DED, associations between sex and the relative abundance of species were different from or even in opposite ways of the trend in healthy individuals. For example, *Pseudomonas aeruginosa* and *Deinococcus* sp. NW-56 were exclusively detected in the samples from female patients with DED (Supplementary Figure S2A, B). However, in the healthy group, *Pseudomonas aeruginosa* was more abundant in males than females (*p* = 0.002), whereas *Deinococcus* sp. NW-56 showed no significant difference in abundance between females and males (*p* = 0.984). Among the 23 microbial species enriched in P-DED, we observed that *Malassezia globosa* was more abundant in female than male patients with DED (*p* = 0.067; Figure 3C). However, for healthy individuals, *M. globosa* was positively associated with male sex (*p* = 0.006; Figure 3D). These results suggest that the sex-related differences in the conjunctival microbiota of patients with DED are distinct from that of healthy individuals.

### Identification of Microbial Species Associated With Different Types of DED

**FIGURE 4:**
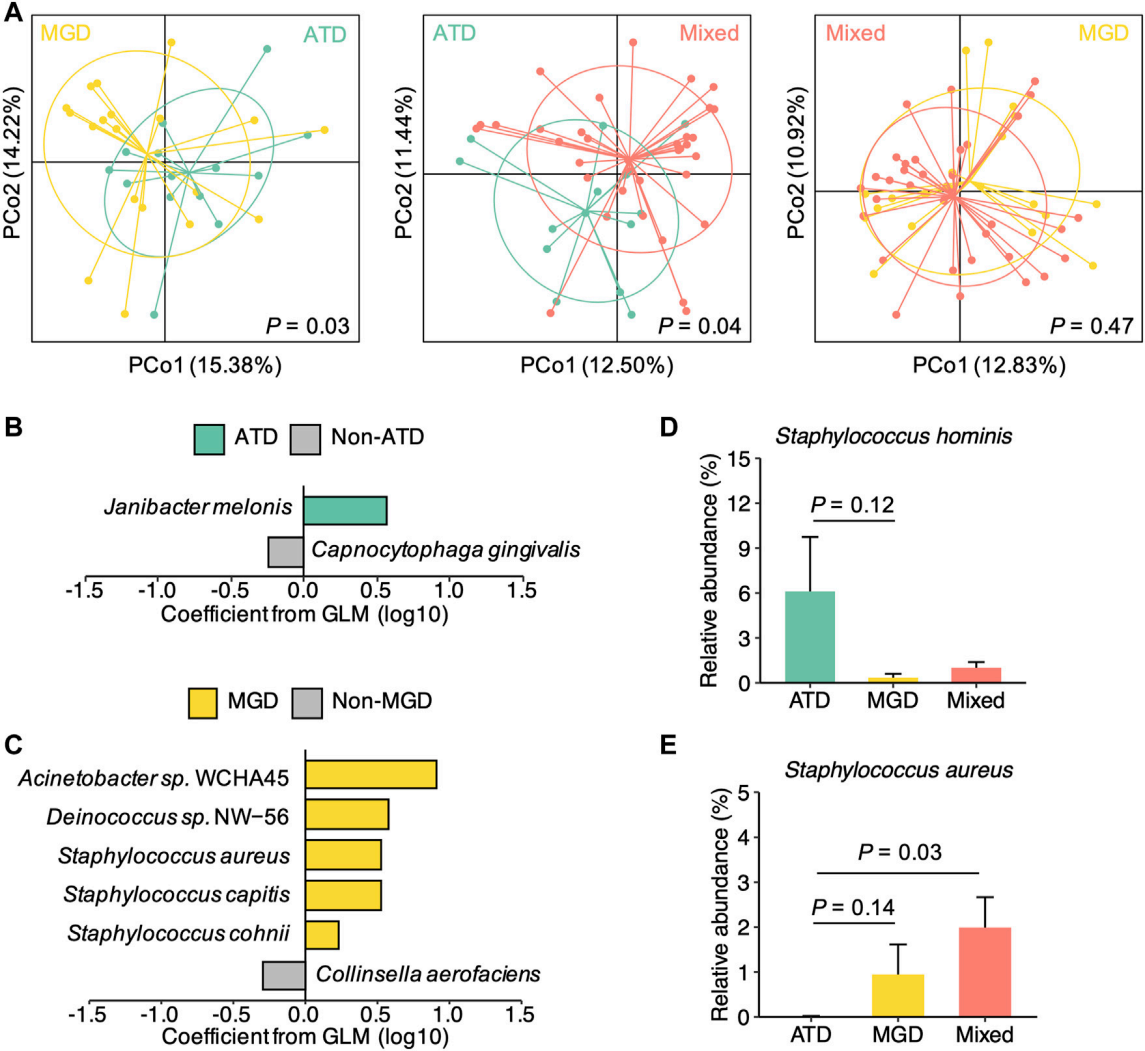
Distinct microbial species signatures of different types of dry eye **(A)** Principal coordinates analysis of the microbial species composition of samples from patients with ATD (*n* = 14) and MGD (*n* = 19), ATD and mixed dry eye (*n* = 35), and MGD and mixed dry eye, respectively. *p* values were computed for PCo1 using Wilcoxon’s rank sum test **(B)** Model coefficients of top-ranked species associated with either ATD or non-ATD dry eye (*p* < 0.1, coefficient >0.2) **(C)** Model coefficients of top-ranked species associated with either MGD or non-MGD dry eye (*p* < 0.1, coefficient >0.2) **(D)** Relative abundances of *Staphylococcus hominis* in the ATD, MGD, and mixed dry eye groups **(E)** Relative abundances of *Staphylococcus aureus* in the ATD, MGD, and mixed dry eye groups. Representative *Staphylococcus* species showing differences in abundance (*p* < 0.15) among the three groups are displayed. *p* values were computed using Wilcoxon’s rank sum test. Relative abundances are represented as mean ± SEM. Error bars indicate standard error.

We next examined the differences in the conjunctival microbiota among patients with different types of dry eye. To improve the representativeness of participants solely with ATD and MGD, 21 additional patients with DED recruited during the same period as the 47 patients were included in our analysis (Supplementary Table S5; Supplementary Figure S3). We first investigated the distribution of samples from patients with ATD, MGD, and mixed dry eye using principal coordinates analysis. We found a delineation between ATD and the other two types of dry eye (MGD and mixed dry eye). In contrast, the delineation between MGD and mixed dry eye were less clear (Figure 4A; Supplementary Figure S4). In agreement with this finding, the microbial diversity in ATD was lower than mixed dry eye (*p* = 0.056). However, the difference in diversity was insignificant between MGD and mixed dry eye (*p* = 0.65; Supplementary Figure S4). These results indicate that the conjunctival microbiota of patients with mixed dry eye is more similar to MGD than ATD.

We hypothesized that different types of DED have distinct species-level signatures of the conjunctival microbiota. To test this hypothesis, we performed multivariable analysis to identify species that were associated with either ATD or MGD. Specifically, the relative abundance of each species was modelled as a function of ATD and MGD as variables and age and sex as covariates. We also examined the overlap between the 23 species with polarized abundance in DED and the species associated with either ATD or MGD. Among the top-ranked associated species (*p* < 0.1, coefficient >0.2), *Janibacter melonis*, which is one of the DED-polarized species, was enriched in ATD (Figure 4B). Moreover, the top three species associated with MGD were all DED-polarized species, including *Acinetobacter* sp. WCHA45, *Deinococcus* sp. NW-56, and *Staphylococcus aureus* (Figure 4C)*.*

We further compared the relative abundance of ATD or MGD associated species among ATD, MGD, and mixed dry eye groups. Notably, species of the genus *Staphylococcus* exhibited different patterns in the three types of DED (Supplementary Figure S5). For instance, *S. hominis* was more abundant in the ATD group than the MGD and mixed dry eye groups (Figure 4D), whereas *S. aureus* was more abundant in the MGD and mixed dry eye groups than the ATD group (Figure 4E). This finding potentially explains the inconsistent observations of associations between *Staphylococcus* and disease status in previous studies that either focused on dry eye or MGD (Gomes et al., 2020). Taken together, these results demonstrate that aqueous deficient, evaporative, and mixed dry eye are associated with distinct microbial species signatures in the conjunctival microbiota.

## Discussion

DED is a multifactorial ocular surface disease whose pathogenesis is not fully understood. Nonetheless, it is recognized that the breakdown of immune homeostasis at the ocular surface plays an important role in the development of DED (Stevenson et al., 2012). Prior studies have revealed the involvement of the ocular microbiota in DED (Gomes et al., 2020). However, conclusions in the alteration of the microbiota can be inconsistent between studies. In this study, we characterized the heterogeneity of conjunctival microbial dysbiosis in DED using data derived from shotgun metagenomic sequencing. We identified 23 species that showed abnormally high abundance in a portion of patients while absent in other patients. Sex is associated with different patterns of microbial dysbiosis in DED. ATD, MGD, and mixed dry eye have distinct signatures of the conjunctival microbiota. The microbial dysbiosis of mixed dry eye is more similar to MGD than ATD.

We observed that the majority of commensal microorganisms in the conjunctival microbiota exhibited a decreased prevalence in DED. Additionally, the α-diversity of patients with DED was lower than that of healthy individuals. These observations are consistent with previous studies (Zilliox et al., 2020; Andersson et al., 2021). Despite the depletion in a portion of DED samples, a group of species exhibited abnormally high abundance in patients with DED compared with healthy individuals. We defined this phenomenon in this study as the polarization in abundance. These findings highlight the heterogeneity of the microbial dysbiosis in DED.

Shotgun metagenomic sequencing enables us to assess the microbial dysbiosis at the species level and survey microorganisms other than bacteria. We detected different patterns of associations with disease status between species of the same genus. These results potentially explained a part of the inconsistent conclusions made at the genus level by previous studies using 16S rRNA sequencing. We believe that strain-level characterization in the future will provide further insight into the heterogeneity of microbial dysbiosis in DED. Furthermore, we identified the polarized abundance of a fungal species *M. globosa*. This species also showed different sex associations between patients with DED and healthy individuals. Therefore, the fungal dysbiosis in DED warrants further investigation.

We found that sex-related differences in the conjunctival microbiota of patients with DED were distinct from that of healthy individuals. Notably, the α-diversity and the abundance of a few microbial species were positively associated with female sex in patients with DED but were positively associated with male sex in healthy individuals. A possible explanation for this inverse trend is that males could tolerate a higher level of perturbations in the conjunctival microbiota than females before the development of DED. Whether the sex differences in the microbiota are associated with a high prevalence of DED in women remains unknown. Furthermore, future studies ideally with sex and age stratification are needed to clarify the associations among sex, DED, and the microbiota.

The conjunctival microbiota in mixed dry eye was more similar to MGD than ATD. An implication of this observation is that the microbial dysbiosis in DED patients with a mixed type of diagnosis is mainly associated with the occurrence of MGD. In agreement with previous studies (Dong et al., 2019), we found that *S. aureus* was positively associated with MGD. We further detected enrichment of *S. aureus* in samples from participants with MGD and mixed dry eye, when compared with ATD samples. This result highlights the involvement of *S. aureus* in MGD. However, the underlying mechanism remains unknown, warranting further investigation.

It has been recognized that DED is a multifactorial disease. According to the TFOS DEWS II definition, tear film instability and hyperosmolarity, ocular surface inflammation and damage, and neurosensory abnormalities can play etiological roles (Craig et al., 2017). The heterogeneity of microbial dysbiosis observed here potentially reflects the complicated etiology of DED. Further research is warranted to explore the link between patterns of microbial dysbiosis and specific etiology of DED. For instance, it may be fruitful to adopt multi-omics approaches integrating data from microbiome and metabolome. Additionally, differences in the microbiota before and after dry eye treatment is worth investigating.

A limitation of this study is that it remains unclear which aspects of the observed microbial dysbiosis are causes or consequences of the onset of DED. Both genetic and environmental factors can influence the conjunctival microbiota. The dysbiosis of microbiota can lead to loss of mucosal tolerance, thereby contributing to inflammation at the ocular surface. On the other hand, loss of homeostasis of the tear film can itself have an effect on the conjunctival microbiota. Furthermore, given the cyclical disease process of DED (Craig et al., 2017), the microbial dysbiosis may be both a cause and a result of DED.

A number of factors may contribute to the inter-individual variation in the microbiota, including host genetic and environmental variables. Importantly, these factors potentially influence both hosts and the microbiota. As a result, it can be difficult to determine whether the microbial dysbiosis mediates the effects of risks factors on the development and exacerbation of DED (Stapleton et al., 2017). To fully characterize the role of the ocular surface microbiota in DED, future studies may perform systemic analysis taking into consideration comprehensive factors like host genetic variants known to affect the composition of the microbiota, lifestyles, and other environmental exposures.

In summary, our study characterized the heterogeneity of conjunctival microbial dysbiosis in DED. It is worth investigating in the future whether microbiota signatures can refine our understanding of DED subtypes. Taken together, our findings offer novel insights into the ocular surface microbial dysbiosis in dry eye and potentially promote the development of microbiota-based personalized strategies for dry eye treatment.

## Data Availability

The data presented in the study are deposited in the Genome Sequence Archive repository, accession number CRA004468.
