# Supplementary material for: Metagenomic Analysis Reveals the Heterogeneity of Conjunctival Microbiota Dysbiosis in Dry Eye Disease
Source: Front Cell Dev Biol. 2021 Nov 25;9:731867. doi: 10.3389/fcell.2021.731867 (PMC8657412; doi:10.3389/fcell.2021.731867)
Supplement: Supplementary file 1 [file Table1.DOCX]

Supplementary Material

# Supplementary Figures and Tables

## Supplementary Figures


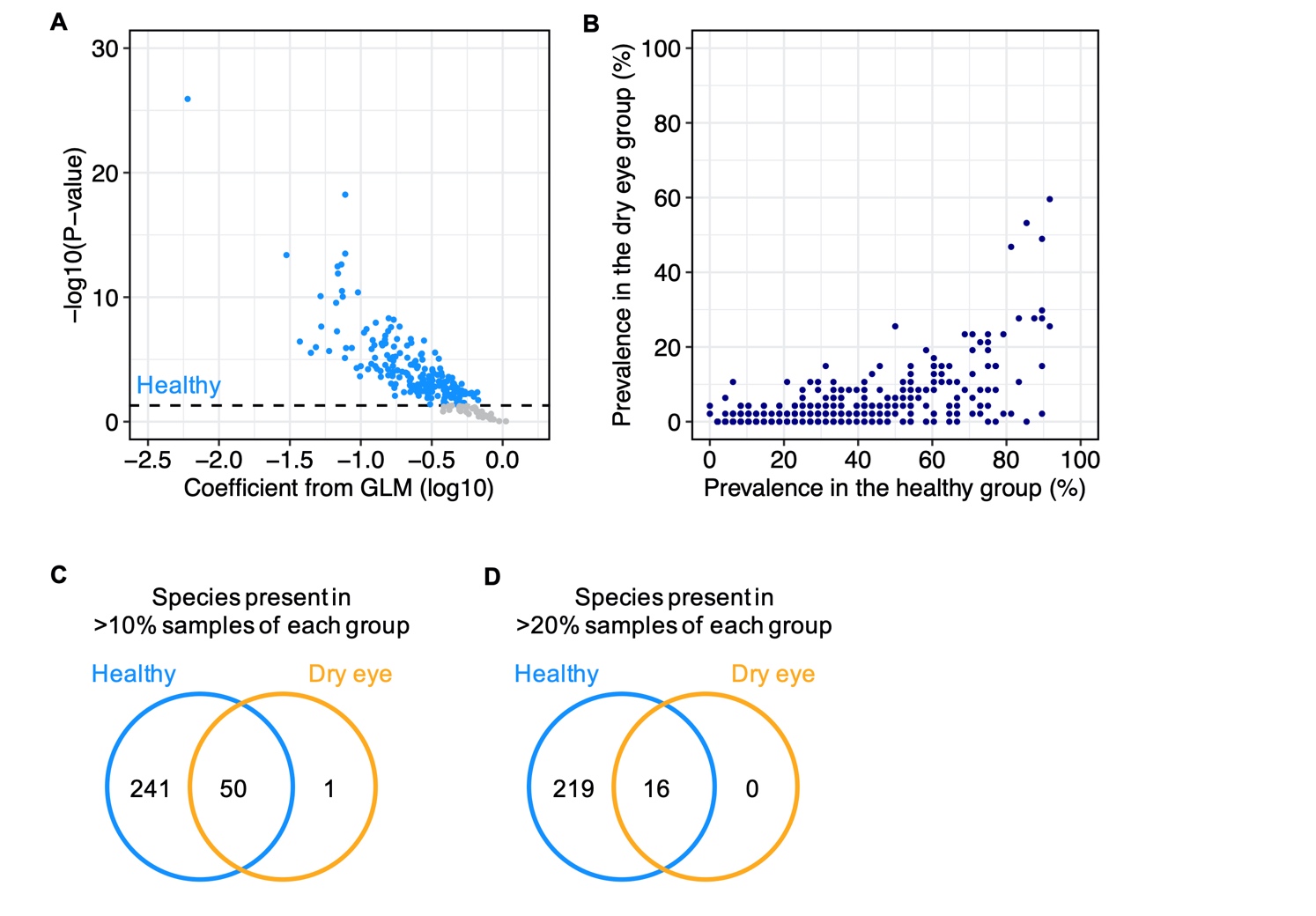


**Supplementary Figure 1.** The conjunctival microbiota in dry eye is characterized by the depletion of commensal microorganisms. **(A)** Volcano plot demonstrating the differential abundance of microbial species between healthy individuals and patients with dry eye. Coefficients and *P* values were derived from general linear models (GLMs). Microbial species are colored if they passed a significance threshold of *P* value < 0.05. **(B)** Prevalence of microbial species in the healthy and dry eye groups, respectively. **(C)** Venn diagram demonstrating the overlap of microbial species present in more than 10% samples between the healthy and dry eye groups. **(D)** Venn diagram demonstrating the overlap of microbial species present in more than 20% samples between the healthy and dry eye groups.

**
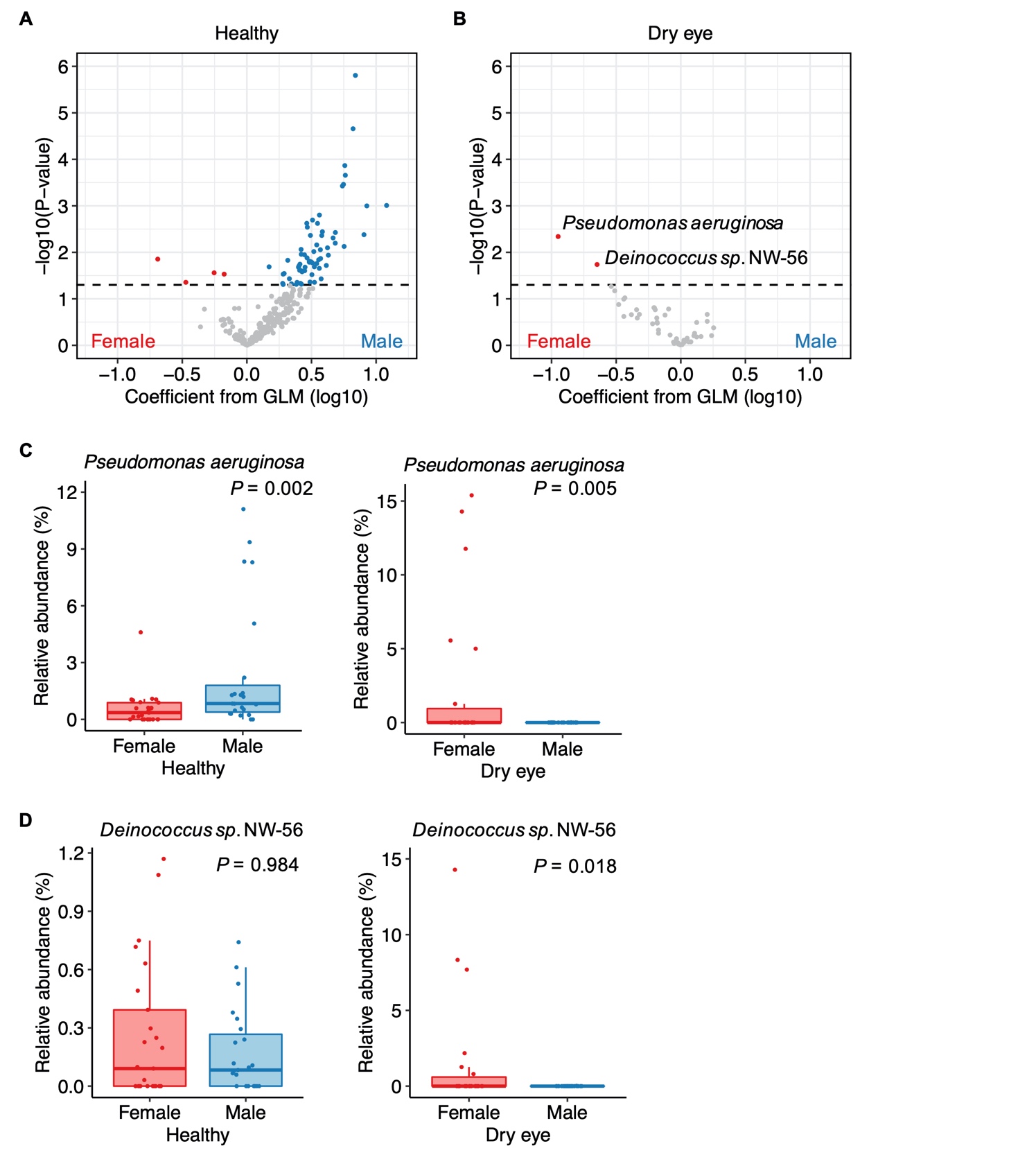
**

**Supplementary Figure 2.** Sex-related differences of the conjunctival microbiota in healthy individuals and patients with dry eye. Volcano plot showing differences in abundance of microbial species between female and male subjects in the healthy group **(A)** and dry eye group **(B)**. Sex-related differences in the relative abundance of *Pseudomonas aeruginosa* **(C)** and *Deinococcus sp.* NW-56 **(D)** in healthy individuals and patients with dry eye. *P* values were derived from multivariable regression adjusting for age.

**
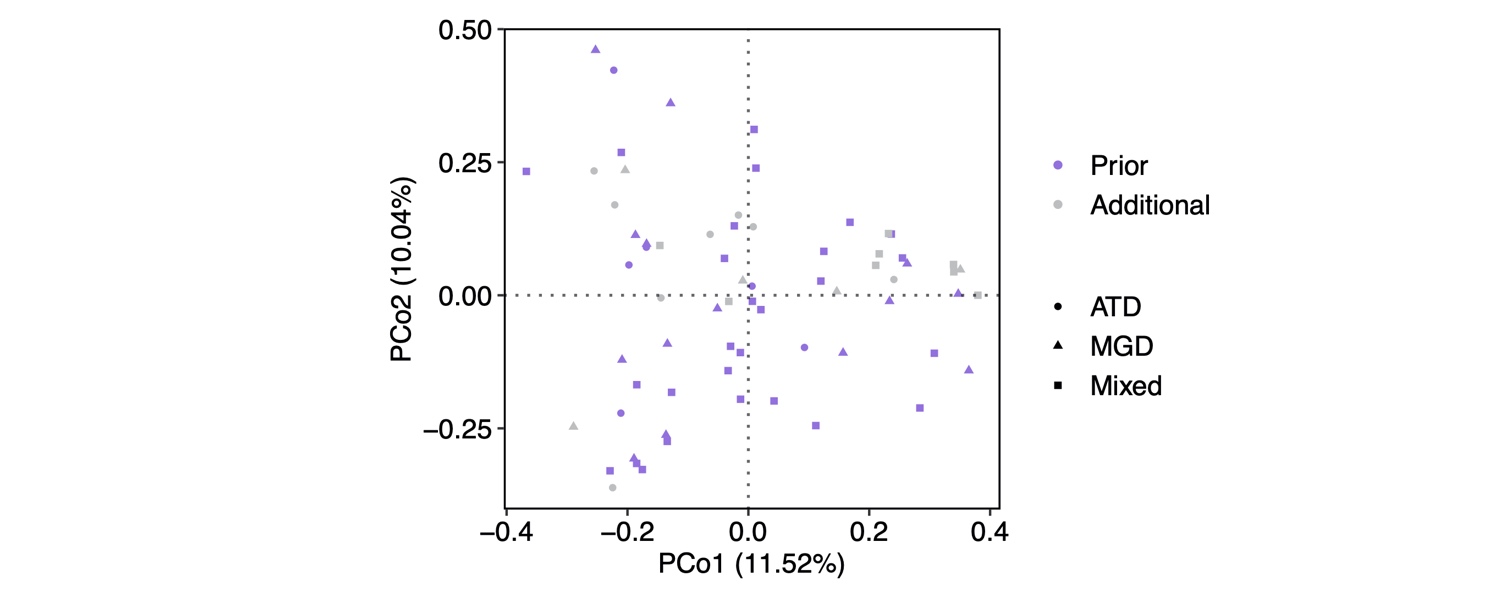
**

**Supplementary Figure 3.** Principal coordinates analysis of the microbial species composition of the conjunctival samples from the prior (*n* = 47) and additional (*n* = 21) groups of patients with dry eye.

**
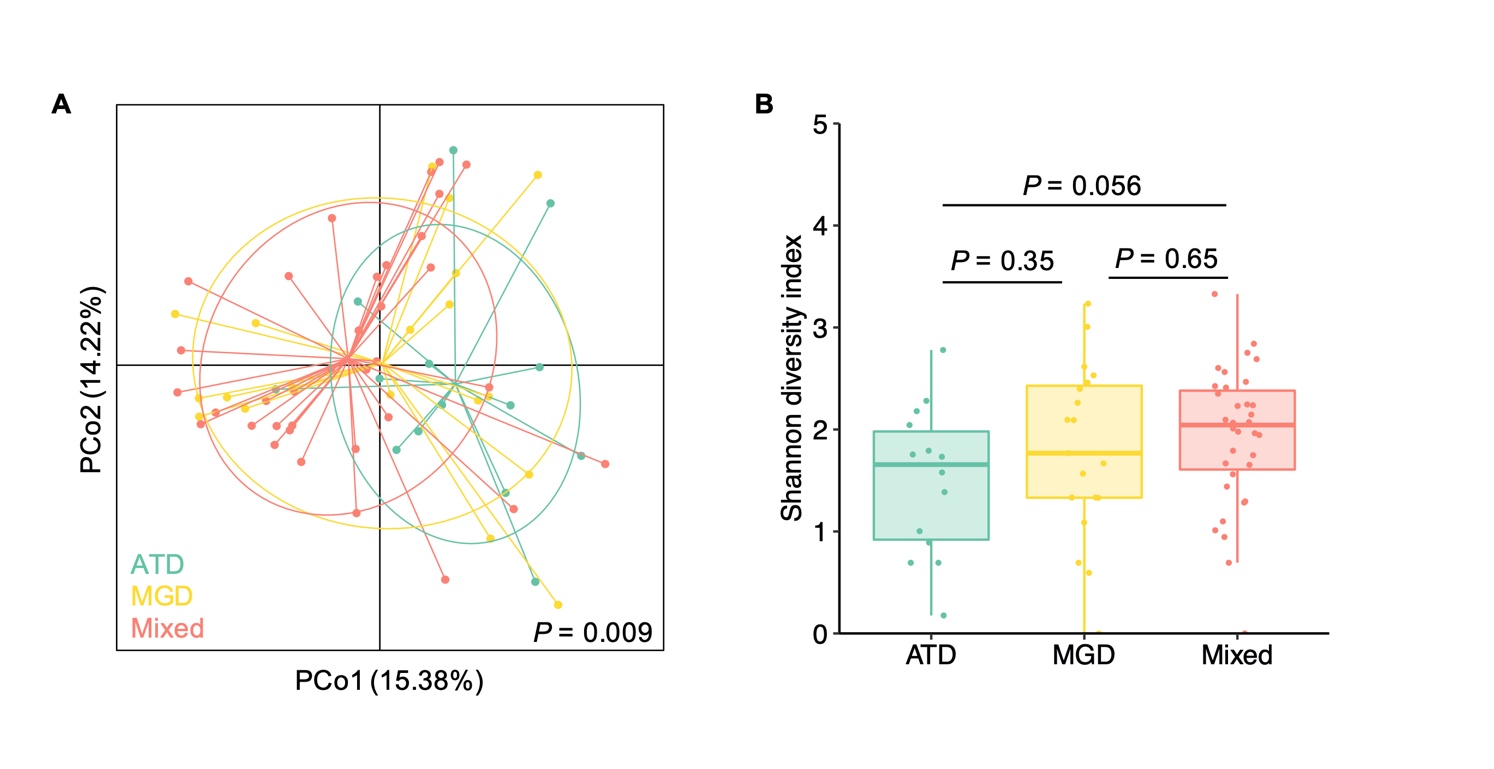
**

**Supplementary Figure 4.** Overall distribution of the conjunctival microbiota samples from patients with different types of dry eye. **(A)** Principal coordinates analysis of the microbial species composition of the conjunctival microbiota in patients with aqueous tear deficiency (ATD, *n* = 14), meibomian gland dysfunction (MGD, *n* = 19), and mixed dry eye (*n* = 35). *P* values were computed for PCo1 using Kruskal-Wallis rank sum test. **(B)** The α-diversity measured with the Shannon index was computed for ATD, MGD, and mixed dry eye samples. *P* values were computed using Wilcoxon’s rank sum test.

**
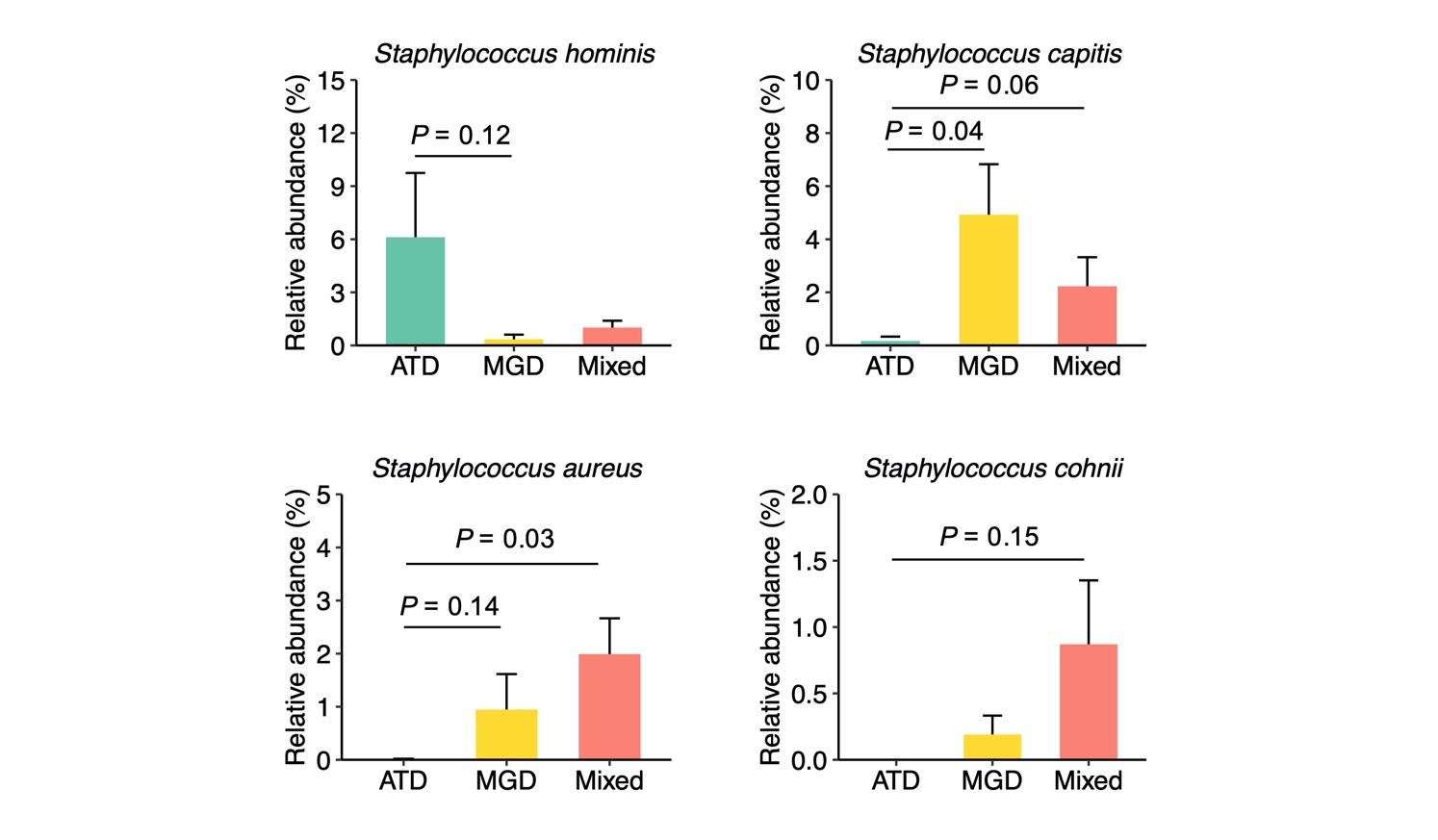
**

**Supplementary Figure 5.** *Staphylococcus* species showing differences in abundance among patients with aqueous tear deficiency (ATD), meibomian gland dysfunction (MGD), and mixed dry eye. The species that were present in at least 10% of the dry eye samples and differentially abundant *(P* < 0.15) among the three groups were shown. *P* values were computed using Wilcoxon’s rank sum test. Relative abundances are represented as mean ± SEM. Error bars indicate standard error.

## Supplementary Tables

**Supplementary Table 1.** Demographic information of healthy and dry eye participants

|  | Healthy | Dry eye |
| --- | --- | --- |
| Number of samples | 48 | 47 |
| Average age (Mean ± SD) | 27.9 ± 4.0 | 40.0 ± 14.6 |
| Sex (Male/female) | 23/25 | 25/22 |

**Supplementary Table 2**. Demographic information of participants with different subtypes of dry eye

|  | ATD | MGD | Mixed |
| --- | --- | --- | --- |
| Number of samples | 6 | 14 | 27 |
| Average age (Mean ± SD) | 29.3 ± 8.6 | 40.9 ± 13.4 | 41.9 ± 15.5 |
| Sex (Male/female) | 4/2 | 6/8 | 15/12 |

ATD, aqueous tear deficiency; MGD, meibomian gland dysfunction; Mixed, patients with both ATD and MGD.

**Supplementary Table 3**. Species significantly contribute to principal coordinate 1 or 2 of dry eye samples

| Species | *P* value (PCo1) | *P* value (PCo2) |
| --- | --- | --- |
| *Acinetobacter sp.* WCHA45 | 3.21E-02 | 7.87E-01 |
| *Aggregatibacter segnis* | 1.87E-03 | 7.01E-02 |
| *Chryseobacterium taklimakanense* | 1.02E-02 | 7.76E-01 |
| *Corynebacterium matruchotii* | 3.31E-03 | 9.11E-01 |
| *Corynebacterium singulare* | 2.16E-02 | 7.59E-01 |
| *Corynebacterium ureicelerivorans* | 1.32E-02 | 9.13E-01 |
| *Cutibacterium avidum* | 1.02E-02 | 9.42E-01 |
| *Cutibacterium granulosum* | 8.23E-02 | 2.62E-07 |
| *Haemophilus parainfluenzae* | 1.75E-04 | 7.63E-01 |
| *Janibacter melonis* | 3.80E-01 | 2.81E-04 |
| *Neisseria subflava* | 2.70E-03 | 5.30E-01 |
| *Parvimonas micra* | 1.08E-04 | 2.51E-02 |
| *Porphyromonas gingivalis* | 5.28E-03 | 3.19E-01 |
| *Prevotella melaninogenica* | 2.48E-05 | 3.19E-01 |
| *Rothia dentocariosa* | 1.62E-02 | 8.40E-01 |
| *Rothia mucilaginosa* | 4.57E-06 | 4.75E-02 |
| *Schaalia odontolytica* | 1.23E-06 | 5.94E-02 |
| *Staphylococcus haemolyticus* | 2.31E-03 | 6.98E-01 |
| *Staphylococcus hominis* | 2.83E-02 | 6.98E-01 |
| *Streptococcus cristatus* | 5.01E-04 | 4.08E-02 |
| *Streptococcus gordonii* | 3.95E-03 | 4.49E-01 |
| *Streptococcus mitis* | 1.09E-04 | 4.20E-01 |
| *Streptococcus oralis* | 6.94E-03 | 4.91E-02 |
| *Streptococcus sanguinis* | 8.46E-04 | 6.59E-02 |
| *Streptococcus sp.* oral taxon 431 | 1.22E-03 | 8.19E-04 |

PCo1, principal coordinate 1; PCo2, principal coordinate 2.

**Supplementary Table 4**. Species with polarized abundance in dry eye samples

| Species | Coefficient (log10) | *P* value | Prevalence  (%, Dry eye) | Prevalence  (%, Healthy) |
| --- | --- | --- | --- | --- |
| *Acinetobacter radioresistens* | 0.85 | 3.03E-02 | 10.6 | 60.4 |
| *Acinetobacter sp.* WCHA45 | 0.83 | 7.66E-06 | 27.7 | 83.3 |
| *Aggregatibacter segnis* | 1.13 | 4.17E-03 | 14.9 | 31.3 |
| *Chryseobacterium haifense* | 0.86 | 5.20E-03 | 14.9 | 75.0 |
| *Chryseobacterium taklimakanense* | 0.96 | 9.86E-03 | 10.6 | 64.6 |
| *Corynebacterium matruchotii* | 0.62 | 1.20E-02 | 23.4 | 70.8 |
| *Cutibacterium granulosum* | 0.54 | 7.69E-04 | 53.2 | 85.4 |
| *Deinococcus ficus* | 1.33 | 8.51E-03 | 10.6 | 29.2 |
| *Deinococcus sp.* NW-56 | 0.71 | 9.48E-03 | 14.9 | 60.4 |
| *Fusobacterium nucleatum* | 1.21 | 4.07E-04 | 10.6 | 52.1 |
| *Janibacter melonis* | 0.40 | 1.93E-02 | 46.8 | 81.3 |
| *Lactobacillus iners* | 1.02 | 1.26E-03 | 14.9 | 60.4 |
| *Malassezia globosa* | 0.71 | 3.55E-05 | 48.9 | 89.6 |
| *Micropruina glycogenica* | 1.19 | 2.93E-05 | 25.5 | 50.0 |
| *Neisseria subflava* | 0.70 | 5.38E-04 | 21.3 | 75.0 |
| *Paracoccus yeei* | 0.65 | 1.17E-02 | 19.1 | 75.0 |
| *Parvimonas micra* | 1.30 | 1.03E-02 | 10.6 | 20.8 |
| *Rothia mucilaginosa* | 0.95 | 5.61E-05 | 23.4 | 68.8 |
| *Staphylococcus aureus* | 0.71 | 3.54E-04 | 23.4 | 75.0 |
| *Staphylococcus hominis* | 0.42 | 2.84E-02 | 27.7 | 87.5 |
| *Streptococcus sanguinis* | 0.77 | 1.49E-02 | 12.8 | 70.8 |
| *Streptococcus sp.* oral taxon 431 | 0.91 | 2.04E-03 | 10.6 | 41.7 |
| *Veillonella parvula* | 0.75 | 6.55E-03 | 19.1 | 70.8 |

Polarized abundance is defined as: (1) prevalence in the dry eye group is lower than the healthy group; (2) relative abundance in the species-positive dry eye samples is significantly higher than healthy samples. Coefficients and *P* values were derived from general linear models adjusting for age and sex.

**Supplementary Table 5**. Demographic information of 21 additional participants with dry eye

|  | ATD | MGD | Mixed |
| --- | --- | --- | --- |
| Number of samples | 8 | 5 | 8 |
| Average age (Mean ± SD) | 44.6 ± 8.6 | 37.6 ± 10.6 | 39.9 ± 17.5 |
| Sex (Male/female) | 1/7 | 4/1 | 5/3 |

ATD, aqueous tear deficiency; MGD, meibomian gland dysfunction; Mixed, patients with both ATD and MGD.
